# mir-145-5p is a suppressor of colorectal cancer at early stage, while promotes colorectal cancer metastasis at late stage through regulating AKT signaling evoked EMT-mediated anoikis

**DOI:** 10.1186/s12885-022-10182-6

**Published:** 2022-11-08

**Authors:** Xianshuo Cheng, Tao Shen, Ping Liu, Shaojun Fang, Zhibin Yang, Yunfeng Li, Jian Dong

**Affiliations:** Department of Colorectal Surgery, The Third Affiliated Hospital of Kunming Medical University, Yunnan Tumor Hospital, Kunming, China

**Keywords:** miRNA 145-5p, Anoikis, EMT, Colorectal cancer

## Abstract

**Background:**

miR-145-5P is generally considered as a tumor suppressor at early stage of colorectal cancer, but up-regulation occurs in the progressive and later stages which is associated with metastasis, indicating miR-145-5p may play dual role in colorectal cancer (CRC). To explore the detailed mechanism of miR-145-5p in carcinogenic is of importance.

**Methods:**

The expression pattern of miR-145-5p in CRC patients was downloaded from TCGA database, and the probable mechanism involved in the carcinogenic effect of miR-145-5p was predicted by bioinformatics analysis. Then, interference of miR-145-5p on SW480 and SW620 cells was conducted, and the influences on tumor cell viability, invasion ability, epithelial-mesenchymal transition (EMT), anoikis, and relative protein expression were examined respectively.

**Results:**

A total of 522 CRC patients’ data indicated that miR-145-5p expression was significantly higher in metastatic CRC than that in non-metastatic CRC, and higher expression of miR-145-5p was correlate with worse prognosis. Overexpression of miR-145-5P-5p enhanced the proliferation and invasion ability of SW620, but inhibited them in SW480. EMT was induced in SW620 after miR-145-5p overexpression and mesenchymal–epithelial transition (MET) was induced in SW480, resulted in the decreased apoptotic rate in SW620 and elevated apoptotic rate in SW480 respectively. Western blot results showed that AKT signaling pathway was involved in the miR-145-5p evoked EMT-mediated anoikis process in SW620 and SW480 cells.

**Conclusion:**

miR-145-5p is a tumor suppressor at early stage of CRC, and an oncogene at advanced stage of CRC. AKT signaling evoked EMT-mediated anoikis might be the pathway by which miR-145-5P regulates CRC cell invasion and metastasis.

**Supplementary Information:**

The online version contains supplementary material available at 10.1186/s12885-022-10182-6.

## Introduction

Colorectal cancer (CRC) is a clonal disease which has a favorable prognosis at localized stage and develops progressively to advanced stages with worse prognosis [1]. To date, CRC is the second leading cause of cancer-related deaths worldwide, and invasion and metastasis are significantly associated with the poor prognosis of CRC [2]. Studies have found that miR-145-5p expression was decreased as early as the pre-adenomatous polyp stage [3], and the downregulation of miR-145-5p was significantly associated with poor overall survival (OS) in CRC [4], suggesting that miR-145-5p may affect pathogenesis of CRC.

miR-145-5p is encoded by miR-145-5P gene which is located on Chromosome 5: 149,430,646–149,430,733 forward strand [5]. This miRNA is mainly considered as a tumor suppressor miRNA in diverse types of cancers, including bladder cancer, breast cancer, cervical cancer, cholangiocarcinoma, renal cancer, and gastrointestinal cancers. In CRC, functional studies have demonstrated that miR-145-5p could suppress CRC migration and invasion, by down-regulating the expression of TWIST1 [6], TUSC3[7], MAPK1 [8], and SIP1 [9], and inhibiting the PAK4-dependent pathway [10]. However, several studies suggested that miR-145 might function as an oncogene. Yuan W et al. reported that miR-145 expression was positively correlated with lymph node metastasis in CRC [11]. Another study showed that miR-145-5p expression was significantly higher in stage III/IV than in stage II CRC patients [12].

Available study indicated that miR-145-5p may play dual roles in CRC, in which it is down-regulated at the early stage as a tumor suppressor, and up-regulation occurs in the progressive and later stages as an oncogene. However, the detailed mechanism of miR-145-5p in carcinogenic has not been fully explained.

Herein, we first revealed the expression pattern of miR-145-5p in CRC patients from TCGA database, and explored the probable mechanism involved in the carcinogenic effect of miR-145-5p by bioinformatics analysis. Then, SW480 cell line which derived from the primary tumor, and SW620 cell line derived from a lymph node metastasis of the same patient at the time of recurrence one year later [13] were selected to examine the role and mechanisms of miR-145-5p in different stages of CRC. Bioinformatics analysis indicated that miR-145-5p expression in metastatic CRC was significantly higher than that in non-metastatic CRC, and higher expression of miR-145-5p was correlate with worse prognosis. miR-145-5p interference results demonstrated that miR-145-5p may promote tumor invasion through AKT signaling driven epithelial-mesenchymal transition-mediated anoikis resistance in SW620, inversely in SW480.

## Materials and methods

### Bioinformatics analysis

Gene expression and corresponding clinical data of CRC, together with adjacent normal mucosa tissues was downloaded from the TCGA platform (https://tcga-data.nci.nih.gov/tcga/). The difference of expression pattern of miR-145-5p between the paired cancer tissues and adjacent normal mucosa tissues was compared first. Then, the relationships of miR-145-5p expression with the clinicopathological characteristics, and the effect of miR-145-5p expression on overall survival of patients were analyzed. Finally, Kyoto Encyclopedia of Genes and Genomes (KEGG) and Gene Ontology (GO) functional enrichment analysis was conducted to explore the potential biological pathway involved in CRC pathogenesis regulated by miR-145-5p expression.

### Cell culture

Human CRC cell lines SW620 and SW480 (American Type Culture Collection, ATCC) were maintained in RPMI 1640 (Invitrogen), supplemented with 10% fetal bovine serum (FBS, Invitrogen) and antibiotics (100 U/mL penicillin and 100 µg/mL streptomycin) in a 5% CO_2_ incubator at 37℃.

### RNA interference of miR-145-5p

miRNA -control, miR-145-5p mimics, miR-145-5p inhibitors -control and miR-145-5p inhibitors were synthesized by Tiangen Biotech (Beijing) Co., Ltd (the sequences were listed in supplemental Table 1). Plasmid was purchased from Tiangen Biotech (Beijing) Co., Ltd. Transient transfections was conducted using Lipofectamine RNAiTM (#C0535, Beyotime). Transfection efficiency was determined by reverse transcription-quantitative PCR (RT-qPCR).

### RT-qPCR

Total RNA was extracted using TRIzol® reagent (Invitrogen; Thermo Fisher Scientific, Inc.), and cDNA was synthesized using Prime Script™ RT Master Mixture (Takara Biotechnology Co., Ltd.). A SYBR Prime Script miRNA RT-PCR kit (Takara Biotechnology Co., Ltd.) was used for the detection and quantitation of miR-145-5p expression. The primers were synthesized by Tiangen Biotech (Beijing) Co., Ltd, and the sequences were as follows: miR-145-5p forward, 5’-CGGTCCAGTTTTCCCAGGA-3’, and reverse, 5’-AGTGCAGGGTCCGAGGTATT-3’. miR-145-5p-loop: 5’-GTCGTATCCAGTGCAGGGTCCGAGGTATTCGCACTGGATACGACAGGGAT-3’.

### Cell viability and invasion assay

To evaluate the influence of RNA transfection on cell viability, cells without interference (WT group), cells transfected with miRNA-control (NC group), and cells transfected with miR-145-5p mimics (OV group) or miR-145-5p inhibitors (IN group) were seeded on 96-well plate at a concentration of 3000 cells/well and cultured as described. Then, cell viability was quantified using a Cell Counting Kit-8 (CCK‐8, Beyotime, China) at 0, 24, 48 and 72 h. The invasion ability of the transfected cells was detected in a Transwell model (8 mm; Millipore). The upper chamber was coated with Matrigel matrix gel (BD Biosciences). Cells (5 × 10^4^ cells/well) resuspended in 200 µl of FBS-free medium were inoculated into the upper chamber, and 600 µl of complete medium containing 10% FBS was added to the lower chamber. After 24 h of incubation, uninvaded cells were removed from the upper surface of the membrane using a cotton swab, and the invaded cells were fixed and stained with 1% crystal violet solution. Finally, light microscopy was performed to photograph and count six random fields of view in each group.

### Immunofluorescence staining

At 48 h after RNA transfection, cells from different groups were seeded onto coverslips in 6-well dishes. After completely adhered to the coverslips, cells were fixed with 4% paraformaldehyde for 30 min, permeabilized with 1% (v/v) Triton X-100 for 10 min at room temperature (RT), and then blocked with 1% goat serum for 1 h at RT. Subsequently, the coverslips were incubated with primary antibodies (anti E-cadherin, Abcam; anti vimentin, Abcam) at 4℃ overnight. Next day, the coverslips were washed three times with PBS and incubated with Cy3- conjugated secondary antibodies (Beyotime) for 1 h at RT, then all the coverslips were counterstained with 4’-6-diamidino-2-phenylindole (DAPI, Sigma-Aldrich). Finally, photomicrographs were captured with a Nikon Total Internal Reflection Fluorescence microscope. The semi-quantitative analysis of immunofluorescence staining was conducted using Image Pro Plus software.

### Anoikis assay by flow cytometry

Cell apoptosis was detected using an Annexin V–FITC Apoptosis Detection Kit (Beyotime) by Flow cytometry according to the manufacturer’s instruction. Briefly, cells from different groups were seeded in Poly-Hema-coated (Sigma-Aldrich) dishes at a concentration of 3 × 10^5^ cells/dish. After 48 h of culture, the cells were harvested and tested in triplicate.

### Western blot

At 48 h after RNA transfection, Cells were lysed using a protein extraction reagent containing protease inhibitor (Beyotime). Total protein concentrations were determined using a BCA protein assay reagent (Beyotime). Then, western blot was implemented as previously described [14]. The primary antibodies used in this study were as follows: mouse anti-human E-cadherin (Abcam), mouse anti-human vimentin (Abcam), rabbit anti-human p-AKT (Ser473) (Abacm), and rabbit anti-human AKT (C67E7) (Cell Signaling Technology) and GAPDH (Beyotime). The semi-quantitative analysis of the western blots was conducted using Image J software.

### Statistical analyses

SPSS 19.0 (SPSS Inc., Chicago, IL) software was used for all the statistical analyses. Results were expressed as mean ± standard deviation (SD). Student’s t test and a one-way analysis of variance (ANOVA) were used for comparison of cell proliferation and invasion between different groups. All experiments concerning cell culture were performed independently at least three times. Potential associations between miR-145-5p expression and the clinicopathological characteristics were evaluated using the chi-square test. Patient overall survival was estimated by the Kaplan-Meier method. *P* < 0.05 was considered statistically significant.

## Results

### High expression of mir-145-5p is associated with metastasis and worse overall survival rates in CRC

**Fig. 1:**
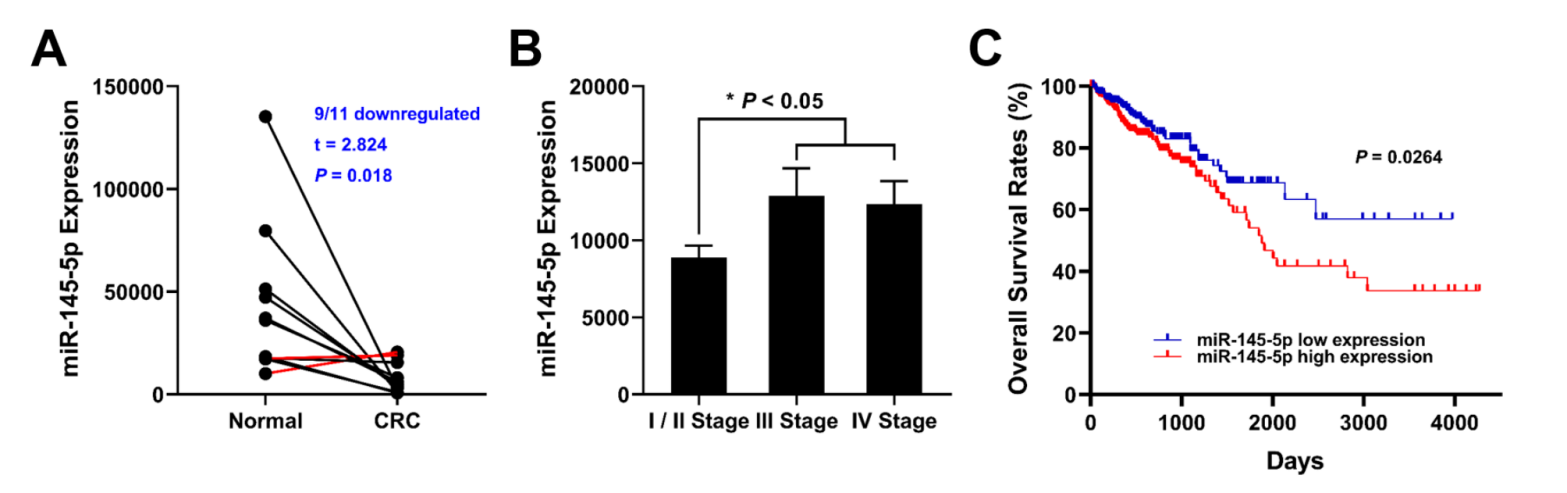
Bioinformatic analysis of 522 CRC tissue data from TCGA. (A) Comparison of miR-145-5p expression between 11 paired CRC tissues and adjacent normal mucosa tissues. (B) Comparison of miR-145-5p expression at different stages of CRC. (C) Comparison of overall survival rates between miR-145-5p low expression and high expression patiens

The RNA-Seq data and corresponding clinical information of 522 CRC tissues, including 11 paired adjacent normal mucosa tissues was collected for bioinformatics analysis. The general information of the collected samples was shown in Table 1, the expression of miR-145-5p was negatively correlated with age (χ2 = 8.955, *P* = 0.003). The comparison results of CRC tissues with paired adjacent normal mucosa tissues suggested miR-145-5p expression was downregulated in CRC (9/11 downregulated, *P* = 0.018 Fig. 1 A). However, the expression of miR-145-5p was significantly higher in CRC with metastasis (including III stage and IV stage) than without metastasis (*P* < 0.05 Fig. 1B), which was in accordance with the results of N stage (χ2 = 7.054, *P* = 0.008), M stage (χ2 = 7.297, *P* = 0.007) and pathological stage (χ2 = 6.983, *P* = 0.008) shown in Table 1. Furthermore, Kaplan-Meier survival analysis indicated that CRC patients with high expression of miR-145-5p had significantly lower overall survival rates (log rank = 4.932, *P* = 0.026 Fig. 1 C).

**Table 1:** Correlation between expression of miR-145-5p and clinical pathological features (Data from TCGA)

	miR-145-5p		X^2^	P*	
	Low expression (n = 261)	High expression (n = 261)			
Sex					
Male(n = 273)	131	142			
Female(n = 249)	130	119	0.929	0.335	
Age					
≥ 60(n = 371)	201	170			
< 60(n = 151)	60	91	8.955	0.003	
Location					
Rectal(n = 140)	66	74			
Colon(n = 382)	195	187	0.625	0.429	
T Stage					
T1 + T2(n = 105)	58	47			
T3 + T4(n = 417)	203	214	1.443	0.23	
N Stage					
Positive(n = 222)	96	126			
Negative(n = 300)	165	135	7.054	0.008	
M Stage					
Positive (n = 87)	32	55			
Negative (n = 435)	229	206	7.297	0.007	
Pathological Stage					
I + II(n = 290)	160	130			
III + IV(n = 232)	101	131	6.983	0.008	

* Chi-square test

### Cell adhesions related pathways are enriched in mir-145-5p high expression CRC

**Fig. 2:**
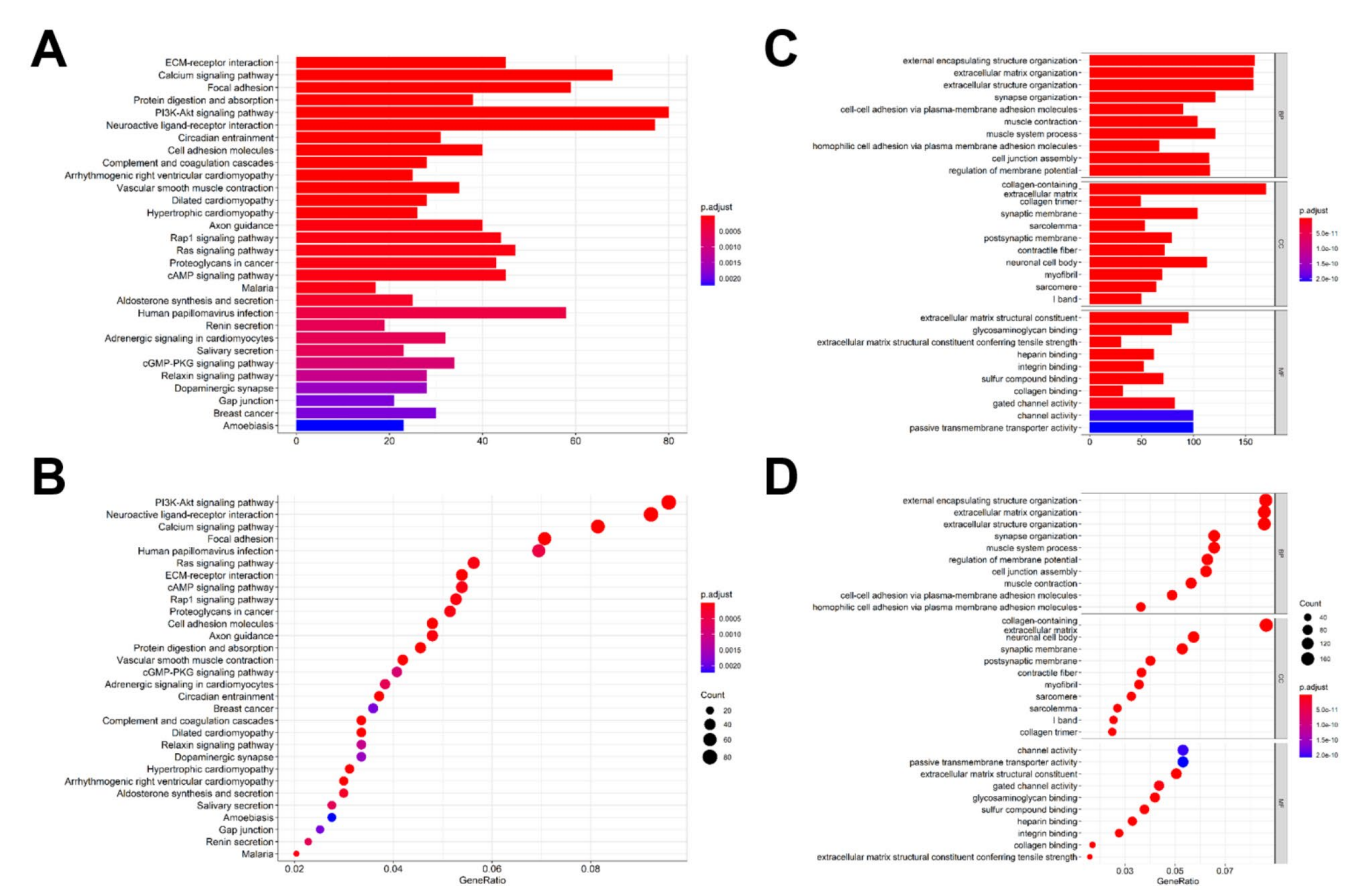
KEGG and GO analyze results. (A) Histogram of KEGG. (B) Bubble chart of KEGG. (C) Histogram of GO. (D) Bubble chart of GO.

In order to clarify the possible mechanism of miR-145-5p in promoting or restricting CRC progression, KEGG and GO analysis was conducted on the collected data. A total of 298 genes were found to express differently between miR-145-5p high expression group and miR-145-5p low expression group, and all the 298 genes were significantly downregulated in miR-145-5p high expression group (Supplementary Table 2). KEGG and GO functional enrichment analysis showed that these differently expressed genes were enriched in 30 pathways, mostly were cell adhesions related pathways, such as KEGG pathways of ECM-receptor interaction, Cell adhesion molecules and Focal adhesions (Fig. 2 A, 2B) and GO terms of cell-cell adhesion via plasma-membrane adhesion molecules, cell junction assembly and extracellular matrix organization (Fig. 2 C, 2D). Additionally, PI3K/AKT signaling was also enriched (Fig. 2B).

### Overexpression of mir-145-5p enhanced the proliferation and invasion ability of SW620, but inversely in SW480

**Fig. 3:**
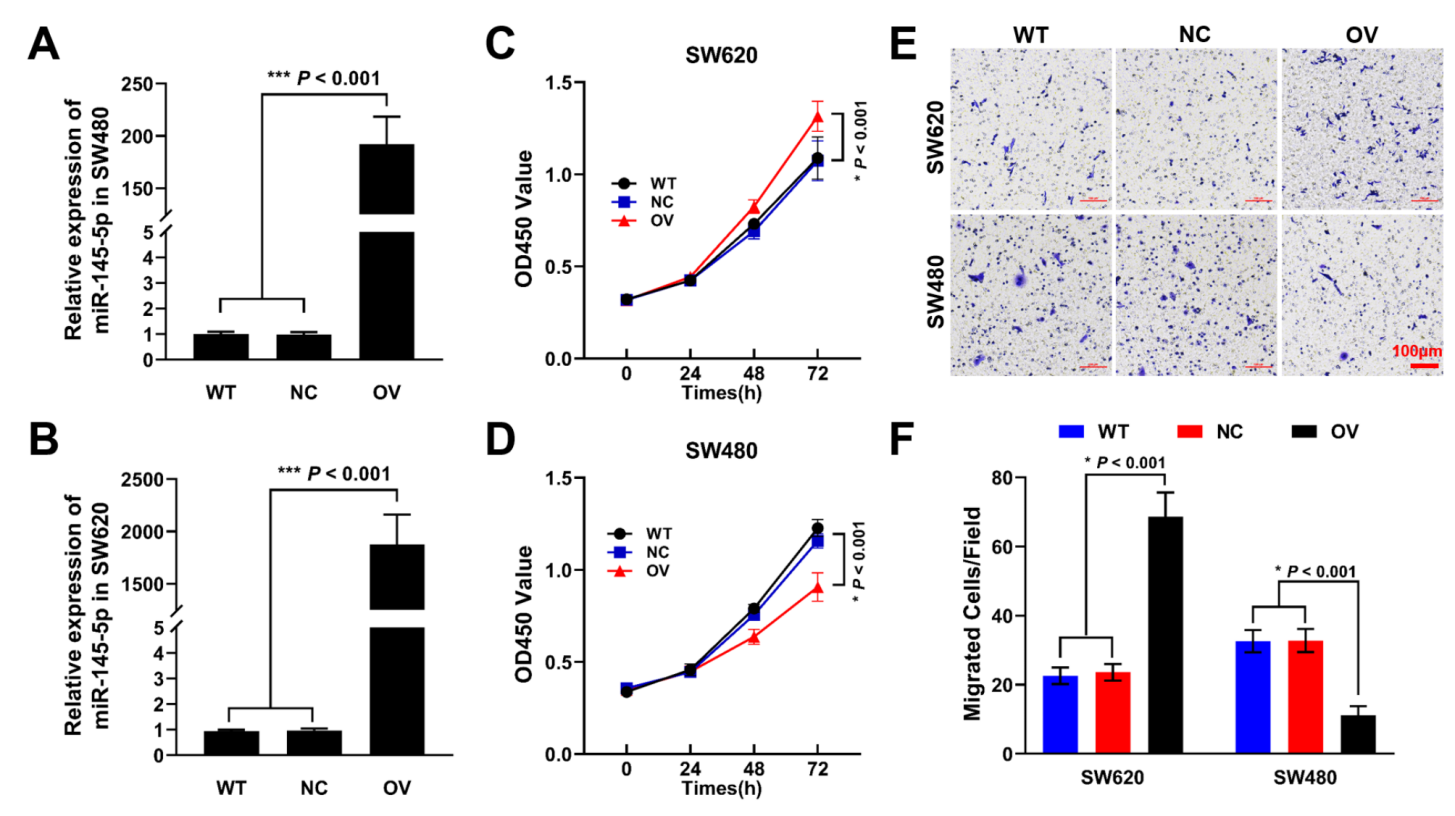
Influence of miR-145-5p on cell viability and invasion ability. The interfering efficiency of miR-145-5p mimics on SW480 (A) and (B) SW620. (C) Influence of miR-145-5p overexpression on SW620 viability. (D) Influence of miR-145-5P overexpression on SW480 viability. (E) Transwell results of SW620 and SW480 after interference with miR-145-5p mimics, scale bar was 100 μm. (F) Calculation of migrated SW620 and SW480 cells according to transwell results

After interference, the transfection efficiency was validated by RT-qPCR at 48 h. As shown in Fig. 3 A, 3B, transfection with miR-145-5p mimics could significantly increase the expression of miR-145-5p in both SW620 and SW480. Furthermore, the CCK-8 results demonstrated that upregulation of miR-145-5p could promote the proliferation of SW620 cells but inhibit it of SW480 cells in a time dependent manner (Fig. 3 C, 3D). Similarly, the overexpression of miR-145-5p could also enhance the invasion ability of SW620 but decrease it of SW480 cells, as indicated by the results of transwell assay (Fig. 3E F).

### Mir-145-5p overexpression induced EMT in SW620 and MET in SW480

**Fig. 4:**
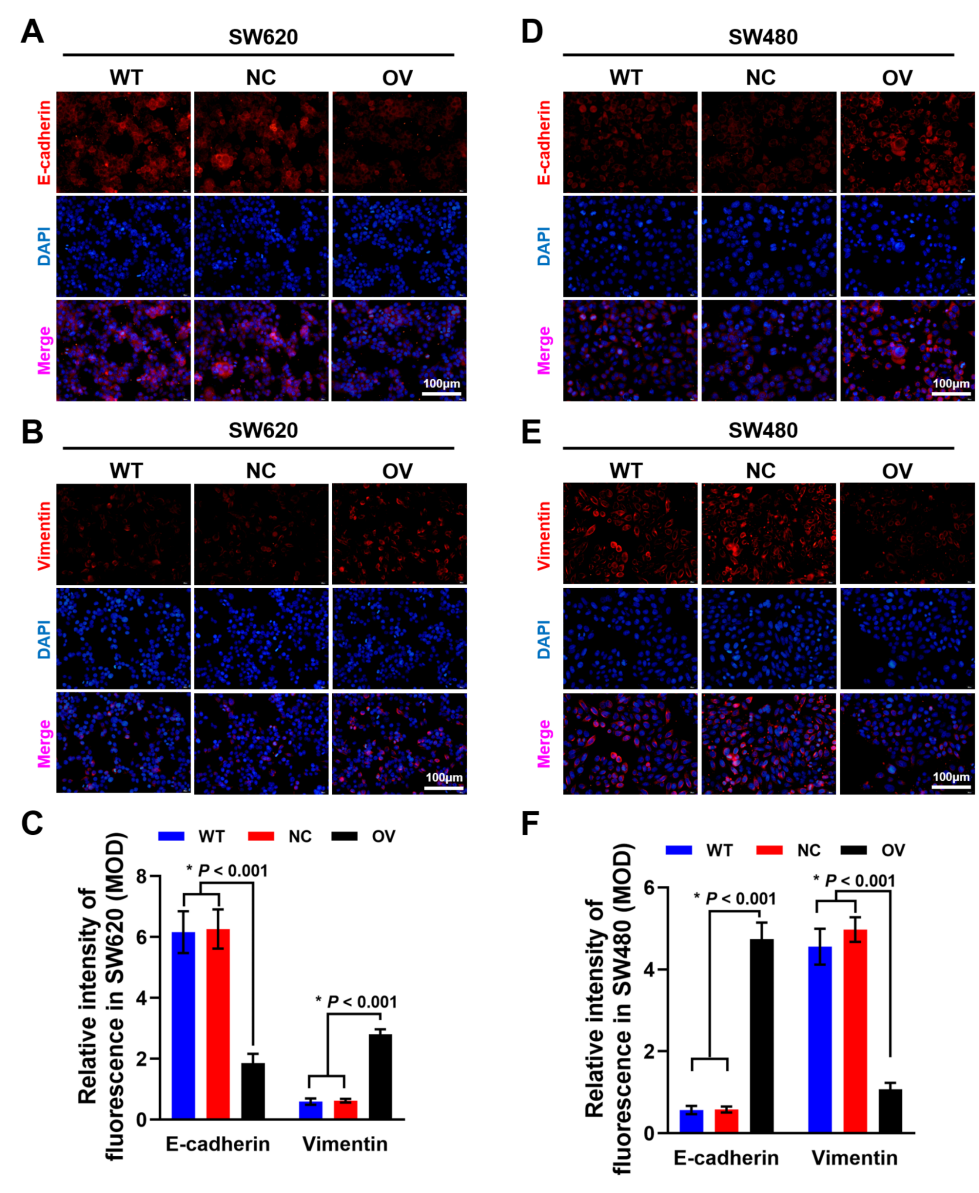
Immunofluorescence staining after RNA transfection. (A) E-cadherin expression in SW620 after miR-145-5p overexpression. (B) Vimentin expression in SW620 after miR-145-5p overexpression. (C) Semi-quantitative analysis results of immunofluorescence staining after miR-145-5p overexpression in SW620. (D) E-cadherin expression in SW480 after miR-145-5p overexpression. (E) Vimentin expression in SW620 after miR-145-5p overexpression. Scale bar was 100 μm. (F) Semi-quantitative analysis results of immunofluorescence staining after miR-145-5p overexpression in SW480.

Immunofluorescence staining was conducted to verify the influence of miR-145-5p on the epithelial–mesenchymal transition (EMT) process of SW620 and SW480 cells, which is a crucial step for the disruption of cell-cell adhesion and cell-ECM interaction. After RNA interference, we found that E-cadherin expression was downregulated (Fig. 4 A) significantly (*P* < 0.001) and vimentin expression was upregulated (Fig. 4B) significantly (*P* < 0.001) in miR-145-5p mimic transfected SW620 cells at 48 h (Fig. 4 C). In SW480 cells, E-cadherin expression was upregulated (Fig. 4D) significantly (*P* < 0.001) and vimentin expression was downregulated (Fig. 4E) significantly (*P* < 0.001) after miR-145-5p mimic transfection, in contrast to that of SW620 cells(Fig. 4 F).

### Mir-145-5p interference had reverse effect on the anoikis of SW620 and SW480 cells

**Fig. 5:**
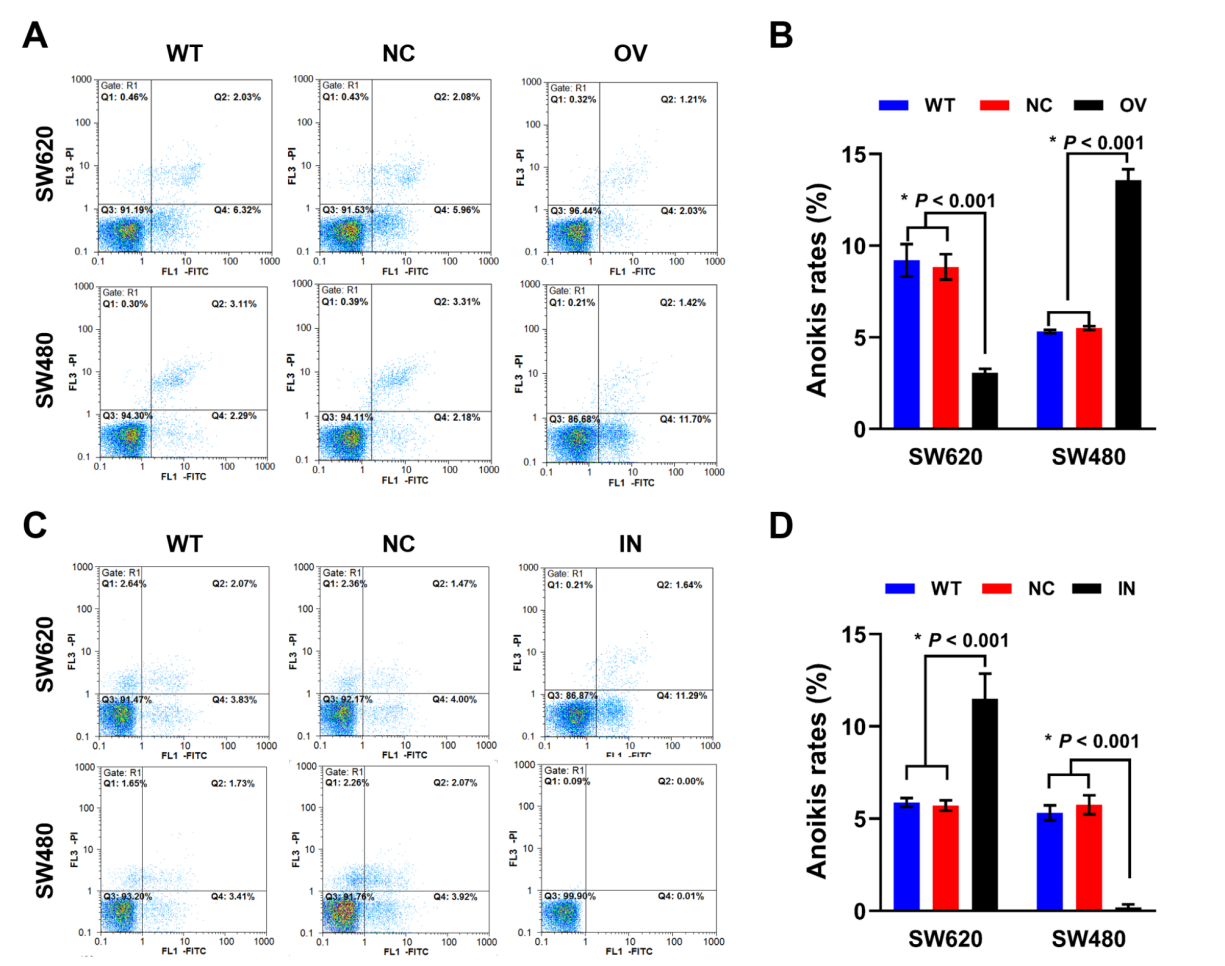
Influence of miR-145-5p on cell anoikis. (A) Flow cytometry results of miR-145-5p overexpression on cell anoikis. (B) Anoikis rates of SW620 and SW480 after miR-145-5p overexpression. (C) Flow cytometry results of miR-145-5p down-regulation on cell anoikis. (D) Anoikis rates of SW620 and SW480 after miR-145-5p down-regulation

After miR-145-5p RNA transfection, cells were suspensively cultured. As shown in Fig. 5, overexpression of miR-145-5p resulted in the significantly decreased apoptotic rate of SW620 cells and significantly elevated apoptotic rate of SW480 cells (Fig. 5 A, 5B). Conversely, after miR-145-5p inhibitor transfection, apoptotic rate of SW620 cells significantly elevated and that of SW480 cells significantly decreased (Fig. 5 C, 5D).

### AKT signaling pathway was involved in the mir-145-5p evoked EMT-mediated anoikis process in SW620 and SW480 cells

**Fig. 6:**
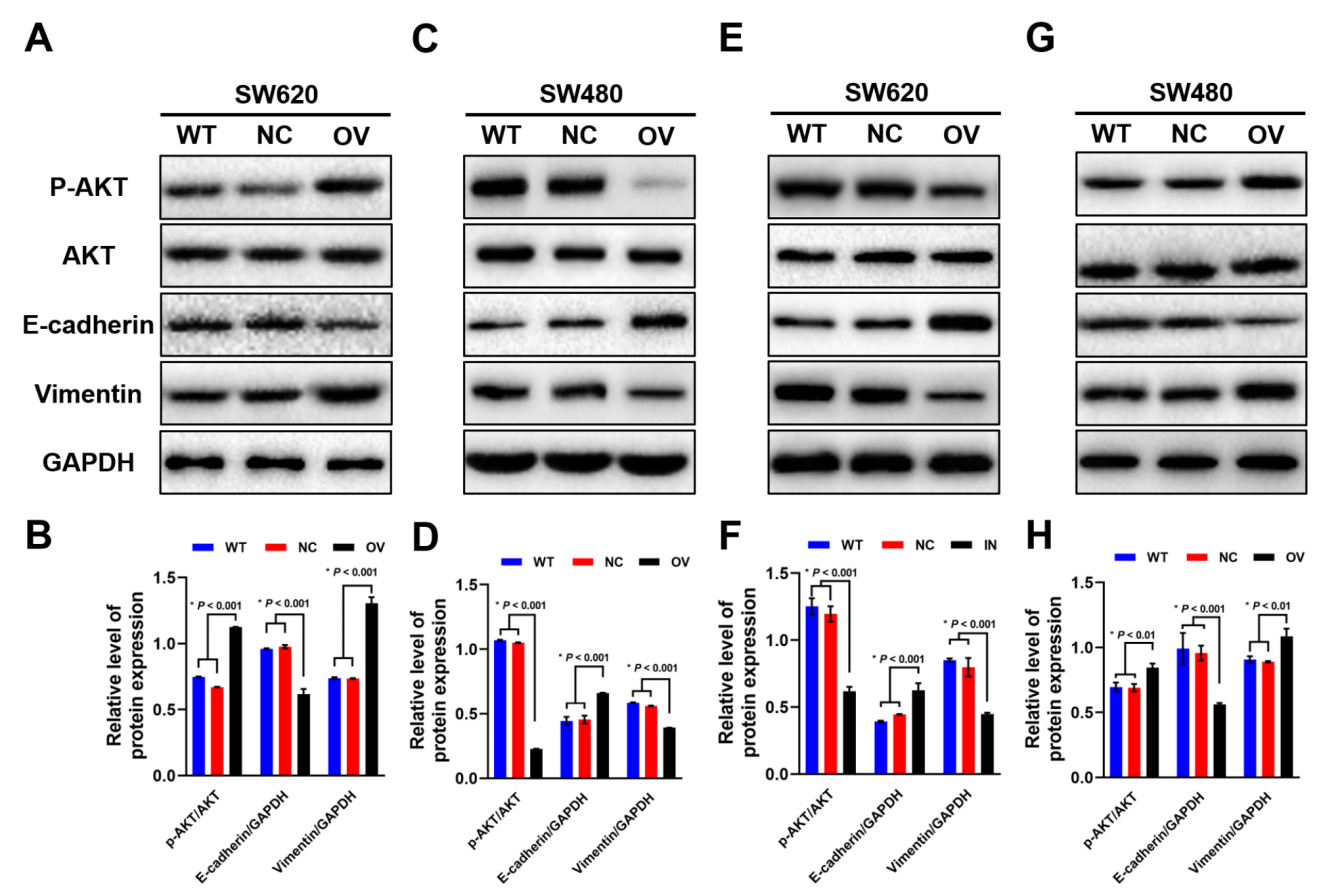
Western blot results of p-AKT, AKT, E-cadherin and Vimentin after miR-145-5p interference. (A, B) Protein expression in SW620 after miR-145-5P overexpression and Semi-quantitative analysis results. (C, D) Protein expression in SW480 after miR-145-5P overexpression and Semi-quantitative analysis results. (E, F) Protein expression in SW620 after miR-145-5P down-regulation and Semi-quantitative analysis results. (G, H) Protein expression in SW480 after miR-145-5P down-regulation and Semi-quantitative analysis results

Since PI3K/AKT pathway was enriched in the bioinformatics analysis, expression of p-AKT and AKT was detected by western blot after miR-145-5p interference, the expression of E-cadherin and Vimentin were also examined simultaneously. In SW620 cells, p-AKT expression was upregulated after miR-145-5p overexpression compared with wild type and normal control. Meanwhile, E-cadherin expression was inhibited and vimentin expression was increased (Fig. 6 A, 6B). The opposite results were observed in SW480 cells after miR-145-5p overexpression, as AKT signaling was suppressed, the expression of E-cadherin was increased and vimentin expression was inhibited respectively (Fig. 6 C, 6D). miR-145-5p inhibitor transfection had opposite effect on SW620 and SW480 cells compared with miR-145-5p mimic transfection. Briefly, p-AKT and vimentin expression was downregulated and E-cadherin expression was upregulated in miR-145-5p inhibitor transfected SW620 cells (Fig. 6E F), while p-AKT and vimentin expression was upregulated and E-cadherin expression was downregulated in miR-145-5p inhibitor transfected SW480 cells (Fig. 6G H). The results suggested that EMT-mediated anoikis process may be correlated with AKT signaling pathway, regulated by miR-145-5p expression in SW620 and SW480 cells.

## Discussion

miRNA is a class of 20–22 nucleotide non-coding RNA molecules that negatively regulate gene expression by inhibiting the translation and stability of target mRNAs [15]. Numerous studies proved that the alteration of miRNA expression is involved in the development of CRC. Among them, miR-145-5p was generally considered to be a tumor suppressor in CRC. By targeting KLF4 [16, 17] and SOX2 [18], miR-145-5p could inhibit the invasion and metastasis of CRC. However, recent study suggested that miR-145-5p exerts oncogenic effect in CRC, as miR-145-5p was upregulated in CRC patients with lymph node or liver metastasis compared to those without metastasis [11]. Hence, it is important to reveal the function of miR-145-5p at different stage of CRC.

In the present study, the expression profiles and clinicopathological characteristics of 522 CRC patients from TCGA database were analyzed firstly. In accordance with previous study, the expression of miR-145-5p in cancer tissues is significantly lower than that in normal mucosa. Besides, higher miR-145-5p expression was observed in stage III/IV CRC tissues than in stage I/II, and worse overall survival rate was accompanied with higher miR-145-5p expression. These finding indicated that down-regulation of miR-145-5p might participate in the initiation of CRC, while re-expression of miR-145-5p might contribute to the deterioration of CRC.

KEGG and GO analyze are valid bioinformatics methods in exploring the potential biological pathway concerned with target gene [19]. The differently expressed genes were mostly enriched in cell adhesions related pathways based on the collected data, which means miR-145-5p might regulate CRC metastasis. EMT is the initial step of cancer invasion, dissemination, and metastasis, during which epithelial cells loss adhesion between tumor cells and the extracellular matrix (ECM) to acquire mesenchymal characteristics and invasive abilities [14, 20]. Accumulating evidence has now indicated that EMT is one of the critical mechanisms that confers cancer cells resistance to anoikis [21–25], then creates a favorable opportunity for tumor proliferation, invasion, and metastasis [26]. It has been demonstrated that PI3K/AKT pathway is a major downstream pathway of miR-145-5p, which is involved in the regulation of invasion and metastasis in different tumor types, mainly in squamous cell carcinoma, bladder cancer and NSCLC[27–29]. AKT activation is a key step for PI3K/AKT signaling pathway to the downstream. Existing evidence has suggested that the activation of AKT is involved in the biological functions of miR-145-5p, such as inhibiting the proliferation of hepatocellular carcinoma [30], inhibiting cardiac fibrosis [31], and promoting the sustained contraction of vascular smooth muscle cells [32]. In CRC, Yin et al. found that miR-145-5p could inhibit AKT activation by targeting N-RAS and IRS1 and suppress VEGF expression in SW116 and HCT116 cells [33].

In the present study, PI3K/AKT signaling pathway was also enriched in the downloaded TCGA data. Further cellular transfection experiments showed that upregulation of miR-145-5p significantly decreased the proliferative and invasive abilities of SW480 accompanied with reduce of p-AKT expression, indicating that miR-145-5p acts as a tumor suppressor in the early stage of CRC. On the contrary, upregulation of miR-145-5p in SW620 significantly increased its proliferative and invasive abilities, and the increase of p-AKT demonstrated that miR-145-5p may act as an oncogene in promoting metastasis.

The dual functions of miR-145-5p in different stage of CRC were verified by transfecting SW620 and SW480 cells with miR-145-5p inhibitor in this study. The flow cytometry and western blot results suggested that AKT signaling evoked EMT-mediated anoikis process was significantly activated in SW620, but suppressed in SW480 cells, when miR-145 expression was inhibited. Interestingly, a previous study reported that miR-145-5p may act as a tumor suppressor in SW620 when using a higher mimic concentration of 50 nM [9]. This might be the main reason that a higher expression of miR-145-5p is positively correlated with metastasis and poor prognosis, although miR-145-5p expression in the cancer tissues is significantly lower than normal mucosa.

The present study had some limitation. Although the results of this study proved that miR-145-5p expressed inversely at different stages of CRC, and AKT signaling evoked EMT-mediated anoikis was regulated by miR-145-5p. The target gene of miR-145-5p through which AKT signaling was regulated was not revealed. Besides, in vivo study was not conducted in this study, which may lead to the less reliability of the conclusion of the current work. Further research based on animal model of CRC was needed to clarify the mechanism of miR-145-5p in regulating CRC progress.

## Conclusion

**Fig. 7:**
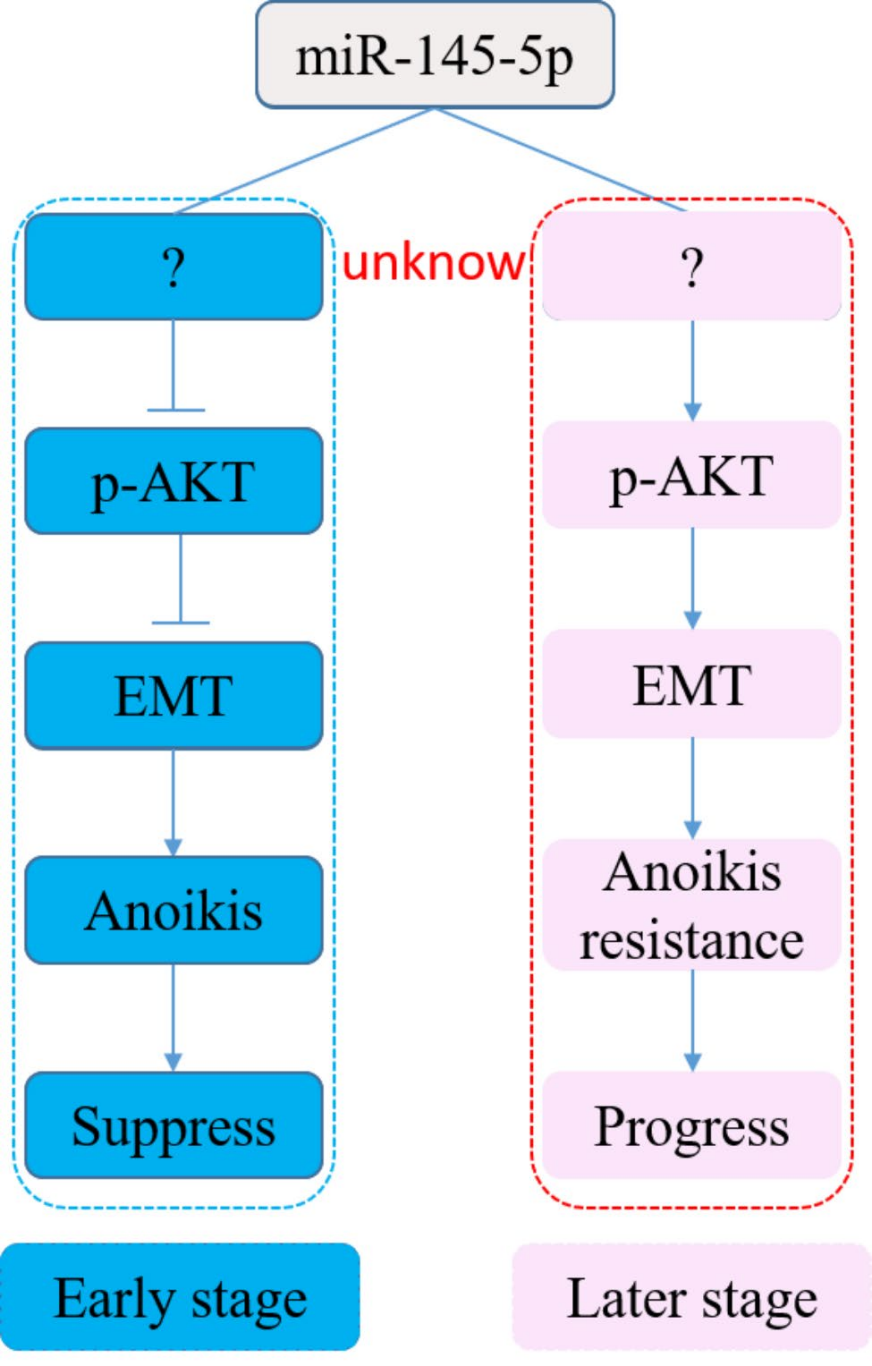
Schematic diagram of the major molecular mechanisms of miR145-5p in regulating CRC progress

The present study consulted to available TCGA data of 522 CRC patients, and found miR-145-5p was down-regulated in CRC, while upswung in III/IV stage with metastasis. Interference of miR-145-5p in SW480 and SW620 cells demonstrated that miR-145-5p played a paradoxical role in the development of CRC, and AKT signaling evoked EMT-mediated anoikis might be the pathway by which miR-145-5p regulates CRC cell invasion and metastasis (Fig. 7). These findings indicated that miR-145-5p was a promising prognostic and predictive markers for CRC in clinical practice.

## Electronic supplementary material

Below is the link to the electronic supplementary material.

Supplementary Material 1

Supplementary Material 2

## Data Availability

The original datasets for bioinformatics analysis in this study can be found in online repositories (https://tcga-data.nci.nih.gov/tcga/). Other datasets used and/or analyzed during the current study are available from the corresponding author or the first author on reasonable request.

## Declarations

### Author details

^1^Department of Colorectal Surgery, The Third Affiliated Hospital of Kunming Medical University / Yunnan Tumor Hospital, Kunming, China.
