# Supplementary material for: mir-145-5p is a suppressor of colorectal cancer at early stage, while promotes colorectal cancer metastasis at late stage through regulating AKT signaling evoked EMT-mediated anoikis
Source: BMC Cancer. 2022 Nov 8;22:1151. doi: 10.1186/s12885-022-10182-6 (PMC9644492; doi:10.1186/s12885-022-10182-6)

miR-145-5p is a Suppressor of Colorectal Cancer at Early Stage, while Promotes Colorectal Cancer Metastasis at Late Stage Through Regulating AKT Signaling Evoked EMT-Mediated Anoikis

running title: miR-145-5p Played a Paradoxical Role in Colorectal Cancer

Authors

Xianshuo Cheng ^1✝^, Tao Shen^1✝^, Ping Liu^1^, Shaojun Fang^1^, ZhibinYang^1^, Yunfeng Li^1^, Jian Dong^1*^

**Original data for WB.** Full-length original images of Western blots showing the effects of miR-145-5p on EMT-related and AKT signaling proteins in SW480 and SW620 cells (in three groups: WT (cells without interference), NC (cells transfected with miRNA-control), and OV (cells transfected with miR-145-5p mimics) or IN (cells transfected with miR-145-5p inhibitors)). Blots surrounded by the red border were corresponding to the Figure 6 in the same order and locations. Blots not surrounded by the red border were experimental replicates or other experiments. The expression of other proteins which had different molecular weight when conducting the WB ECL-HRP detection process should be detected for other researching purpose. Thus, to avoid acquiring multiple protein bands in one field, which would interfere the results presentation of this study, protein bands which considered to be interest protein were cut prior to hybridisation with antibodies according to the hint of protein marker.


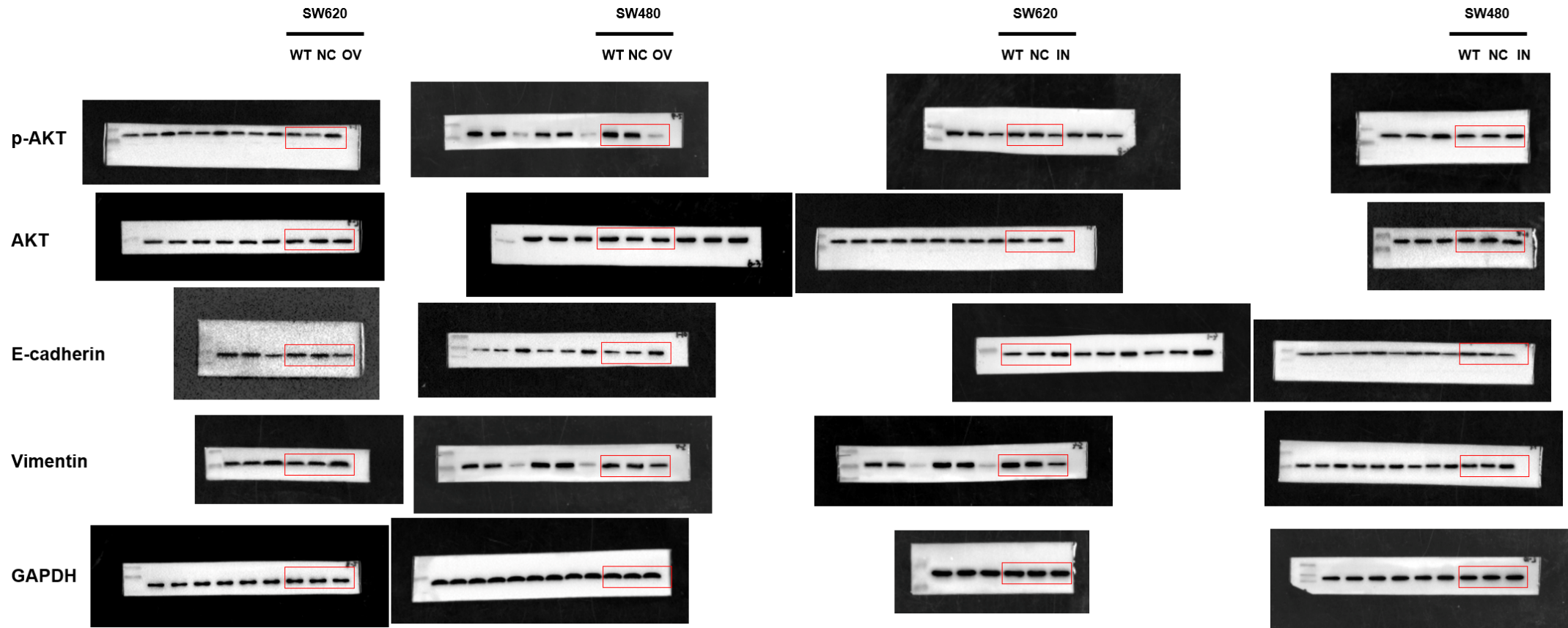

Supplement: Supplementary file 2 — Supplementary Material 2 [file 12885_2022_10182_MOESM2_ESM.docx]
